# High performance solutions and data for nZEBs offices located in warm climates

**DOI:** 10.1016/j.dib.2015.09.041

**Published:** 2015-10-13

**Authors:** Paolo Maria Congedo, Cristina Baglivo, Ilaria Zacà, Delia D׳Agostino

**Affiliations:** a Department of Engineering for Innovation, University of Salento, 73100 Lecce, Italy; b Energy efficiency and Renewables Unit, Institute for Energy and Transport (IET), Joint Research Centre (JRC) – European Commission, Ispra, VA, Italy

**Keywords:** ZEB, EPBD, Building, Efficiency, Office, Renewables, Cost-optimality

## Abstract

This data article contains eleven tables supporting the research article entitled: Cost-Optimal Design For Nearly Zero Energy Office Buildings Located In Warm Climates [1].

The data explain the procedure of minimum energy performance requirements presented by the European Directive (EPBD) [2] to establish several variants of energy efficiency measures with the integration of renewable energy sources in order to reach nZEBs (nearly zero energy buildings) by 2020.

This files include the application of comparative methodological framework and give the cost-optimal solutions for non-residential building located in Southern Italy. The data describe office sector in which direct the current European policies and investments [3], [4].

In particular, the localization of the building, geometrical features, thermal properties of the envelope and technical systems for HVAC are reported in the first sections. Energy efficiency measures related to orientation, walls, windows, heating, cooling, dhw and RES are given in the second part of the group; this data article provides 256 combinations for a financial and macroeconomic analysis.

**Specifications Table**

**Table:** 

Subject area	*Civil engineering*
More specific subject area	*High energy efficiency measures for non-residential buildings*
Type of data	*Tables*
How data was acquired	*ENEA reports*[3], [4]*, technical datasheets*[5]*, Puglia price list, market surveys, ISTAT surveys, software ProCasaClima 2015*
Data format	*xls*
Experimental factors	*No pretreatment of samples was performed*
Experimental features	*Cost-optimal solutions have been derived for several combinations of energy efficiency technical variants, applied to a non-residential reference building located in Southern Italy.*
Data source location	*Lecce – Italy, Mediterranean climate*
Data accessibility	*Data is provided in Supplementary materials directly with this article*

**Value of the data**

• Identification of 256 combinations of energy efficiency measures for office buildings.
• Assessment of optimal energy solutions in terms of primary energy consumptions and global costs for non-residential buildings.
• Methodological approach to understand and exploit the possible solutions for nZEBs in warm climate.

## 1 Data

Eleven tables are provided in order to give the input (geometrical features, thermal properties of external walls and windows, characteristics of the technical systems and investment costs) and output data (primary energy consumptions, CO_2_ gas emissions, global costs) of non-residential buildings located in warm climate (Southern Italy, Lecce).

## 2 Experimental design, materials and methods

The design of cost-optimal nZEB has been carried out for new office buildings located in Southern Italy through the implementation of several steps. The main pillars of the methodology include the definition of the reference building, the identification of energy efficiency measures establishing the combinations of all variants and assessing the energy performance and the global costs with sensitivity analysis.

The data are derived for a building located in warm climate (Lecce, Italy, climatic zone C) having 1153 degree-days. The country is characterized of a non-extreme winter, but high aridity in summer and rainfall concentered in autumn and winter [6], [7], [8], [9]. In other Member States as well as in the islands of Italy, is very common this type of climate, but not previously considered for this kind of non-residential building.

The input data about the geometrical features (perimeter, area, volume, shape factor, etc.) are derived from ENEA reports [3], [4] that apply the methodology to a reference building in accordance with [10] representing the typical building stock in a country. The files include the data of the smallest office building in the reports, although materials and systems represent the ones commonly used in the country. The tables in sheets S1a and b, in the .xls file in attachment, give details about the architectural features, building type descriptions and construction elements properties. The building has the internal heat gains including 32 employees, equipment and lighting systems.

The aim of the research is to reduce the energy demands and the primary energy consumptions for office buildings in order to reach nZEBs level by applying several combinations of variants. The measures proposed include different type of walls, windows and technical systems (for heating, cooling, ventilation, generation and RES). The selected highly external precast walls and the type of windows frame are reported in the Tables S2a and b; the data of the external walls are derived from the integration of a multi-criteria optimization analysis carried out in mode FRONTIER rel.4.3 environment with calculation procedures to evaluate the dynamic performance of building components developed in MatLab rel.7.0 environment [11], [12]. For the thermal properties of the windows, the output values have been obtained by implementing directly in the software the size, the solar factor, the conductivity of glazing and frame.

Tables S3a and b show all the variants related to technical systems, including investment costs. The main characteristics of generations, emissions, and ventilations, have been derived from technical sheets.

The software ProCasaClima2015 has been used for the evaluation of the energy demands, the primary energy consumptions and the global costs for all combinations, according with [13], [14], [15], [16], [17]. Tables S4a and b include the values of energy demands for monthly and annual assessment. All the 256 combinations obtained are shown in Table S5, where the output values of CO_2_ gas emissions, primary energy consumptions, global costs and the performance classification are listed in terms of financial analysis. Macroeconomic output values are given only for the best range of solutions (Table S6).

The analysis led to define seven ranges of primary energy consumptions. Table S7 shows the quality of each interval by the different colors related to the primary energy consumptions and CO_2_ gas emissions ranges. Each one is characterized by a combination of specific technical system variants.

The study contributes to define the strategies to reach high performance energy buildings for Mediterranean climate according with the national requirements. The reduction of CO_2_ gas emissions, primary energy consumptions and costs have been obtained for several solutions compared to the reference building.

## Author contributions

All authors participated in preparing the research from the beginning to ends, such as establishing research design, method and analysis. All authors discussed and finalized the analysis results to prepare manuscript according to the progress of research.

## Footnotes

Appendix A Supplementary data associated with this article can be found in the online version at doi:10.1016/j.dib.2015.09.041.
